# Drain Current Modulation of a Single Drain MOSFET by Lorentz Force for Magnetic Sensing Application

**DOI:** 10.3390/s16091389

**Published:** 2016-08-30

**Authors:** Prasenjit Chatterjee, Hwang-Cherng Chow, Wu-Shiung Feng

**Affiliations:** Graduate Institute of Electronic Engineering, Chang Gung University, 259 Wen-Hwa 1st Road, Kwei-Shan, Tao-Yuan 333, Taiwan; fengws@mail.cgu.edu.tw

**Keywords:** MOSFET, magnetic sensor, Lorentz force

## Abstract

This paper reports a detailed analysis of the drain current modulation of a single-drain normal-gate n channel metal-oxide semiconductor field effect transistor (n-MOSFET) under an on-chip magnetic field. A single-drain n-MOSFET has been fabricated and placed in the center of a square-shaped metal loop which generates the on-chip magnetic field. The proposed device designed is much smaller in size with respect to the metal loop, which ensures that the generated magnetic field is approximately uniform. The change of drain current and change of bulk current per micron device width has been measured. The result shows that the difference drain current is about 145 µA for the maximum applied magnetic field. Such changes occur from the applied Lorentz force to push out the carriers from the channel. Based on the drain current difference, the change in effective mobility has been detected up to 4.227%. Furthermore, a detailed investigation reveals that the device behavior is quite different in subthreshold and saturation region. A change of 50.24 µA bulk current has also been measured. Finally, the device has been verified for use as a magnetic sensor with sensitivity 4.084% (29.6 T^−1^), which is very effective as compared to other previously reported works for a single device.

## 1. Introduction

Magnetic sensors have been used extensively in different fields like vehicle detection systems and biosensing applications [1,2,3,4]. A variety of magnetic sensors for sensing moderate or lower magnetic fields at low cost are available viz. split-drain magnetic field effect transistor (MAGFET), Hall plate, etc. The physical phenomenon of carrier deflection of moving charges by the application of magnetic field in the inversion layer of the MOSFET has been exploited initially in [5,6,7]. The applied magnetic field perpendicular to the inversion layer can be sensed by an imbalance of currents or the Hall voltage depending on the design of the sensing device. This approach does not interfere the electrical characteristic of the MOSFET, as reported in [6]. This kind of sensors can be batch-fabricated at very low cost and is able to detect the low magnetic field at room temperature.

As an another alternative to using the MOSFET inversion layer as the active area for magnetic field sensing applications, the drain of the traditional MOSFET can be equally divided into two or three parts [8,9,10,11,12,13,14]. These drains share equal amounts of current under no influence of the magnetic fields. In the presence of the magnetic field across the inversion layer of the split-drain MOSFET (MAGFET), an imbalance in current can be detected between these split-drains, as the created Lorentz force deflect carriers in the channel and this current imbalance is proportional to the applied magnetic field. From [12] it can be noted that the current imbalance is related to the deflection parameters including channel length, mobility of the charged carriers, and the magnetic field strength. Although, to get the equal currents in two drains the design of the sensor must be very accurate geometrically, otherwise a large amount of offset current will degrade the sensor performance. To overcome this offset error, complex design techniques must be implemented viz. chopping spinning technique [15] or offset-trimmable array [16]. Also, the MAGFET structure is prone to noise-related issues that noticeably reduce the resolution of the sensors.

In another approach, Wakiya et al. [17] proposed drain current modification by the magnetic field, but the device in that case is a Pt/MZF/YSZ NMOS device. As reported in [17], the device offers a small amount (order of pA) of the drain current increment due to the vertically applied magnetic field (i.e., the drain current modification) by increasing the mobility of the electrons. Simultaneously, the applied magnetic field pushes the carries away from the channel which decreases the mobility—such comments are self-contradictory.

Gabara [18] reported the creation of a magnetic field by on-chip metal loops, however, in this work, the proper characterization of the device has been ignored, and instead limited to oscillation frequency.

Therefore, all the previous works either focused on Hall effect measurements, MAGFETs, or on the oscillation frequency measurement instead of device characterization. Therefore, the drain current modulation by the very small on-chip magnetic field for a MOSFET device with single-drain structure remains unexplored. In this paper, the detailed characterization of drain current in n-MOSFET has been carried out. The magnetic field for this work has been generated by an on-chip metal loop, which leads to a deflection of the charged carriers in the channel region and, as a result, a significant amount of drain current difference can be observed. This change in drain current ascribed to change in mobility, and the change in mobility is also calculated from the measured drain current. The change in substrate current can also be observed and is shown in the following sections. The change in drain current due to the applied magnetic field in n-MOSFET can lead us to the conclusion that this single-drain normal-gate n-MOSFET can be used as a potential magnetic sensor.

## 2. Device Structure and Principle

**Figure 1:**
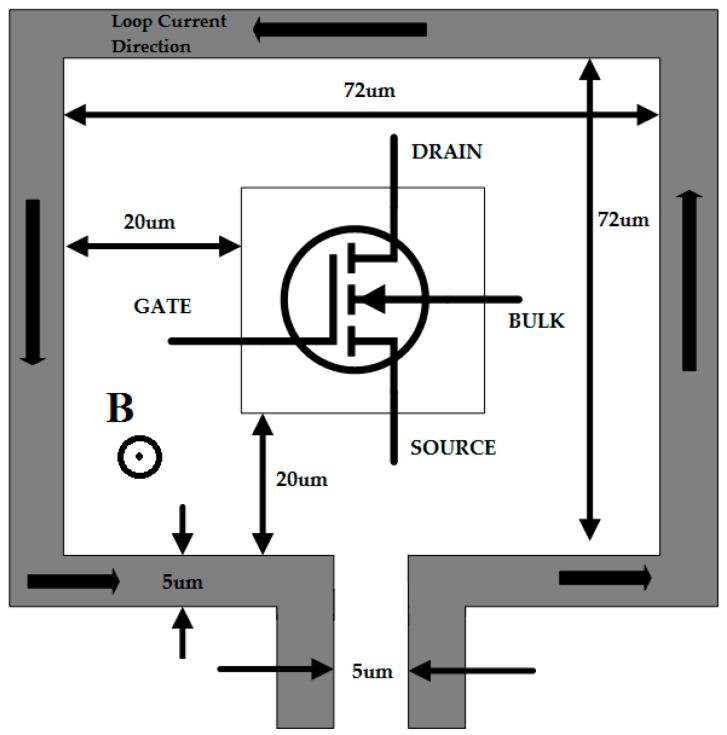
Schematic diagram of the device under test.

The basic principle of this structure is based on the effect of the Lorentz force on mobile electrical charges under the presence of a steady magnetic field. In Figure 1, the four terminals of the transistor are marked as gate, bulk, drain, and source, respectively; the arrows on the metal strip indicate the direction of the current flow; and B is the generated magnetic flux. The metal loop is square in shape and each side is 72 µm in length. The width of the metal loop is 5 µm and the gap is also 5 µm to open the contacts. The force created due to the magnetic flux equals to

(1) F=−q (E+v×B)

where *q* represents the charge of the mobile carrier, ***E*** is the electric field, and ***ν*** is the velocity of the majority carriers. The loop metallization is implemented in close proximity to the surface active area, which ensures the maximum effect of magnetic field strength on the device active region. The magnetic flux ***B*** can be approximated by [19]

(2) B=μ I L22π (x2+L24) √(x2+L22)

where *I*, is the current through the metal loop, *L* represents the length of each side of the metal loop, *x* depicts the distance from the top of the device in the center of the loop, and *µ* is the permeability.

**Figure 2:**
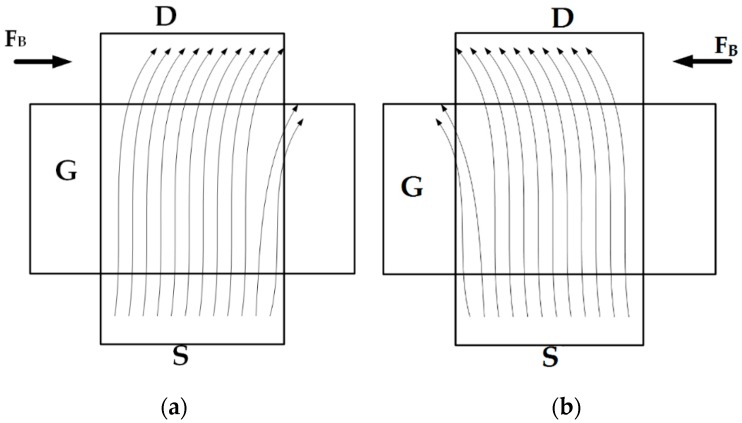
Illustration of carrier deflection under magnetic field. (**a**) With forward loop current; (**b**) with reverse loop current.

The force generated by *F_B_* will push charged carriers in the channel and hence a current difference will occur. This can be explained according to Figure 2.

Under normal circumstances all the carriers will reach to the drain, but due to F_B_ (as shown in Figure 2a) carriers will push out of the channel, and hence the effective drain current will decrease. If the current direction through the loop is inverted (opposite to that of the Figure 1) then the effect will be like Figure 2b. In this case also F_B_ pushes the carrier out of the channel, and the effective drain current will decrease. As F_B_ pushes away the carriers from the channel, the inversion layer charge will reduce and the drain current will be reduced. The aforementioned explanation can be verified by use of measurement results in the next section.

## 3. Experimental Results and Discussions

**Figure 3:**
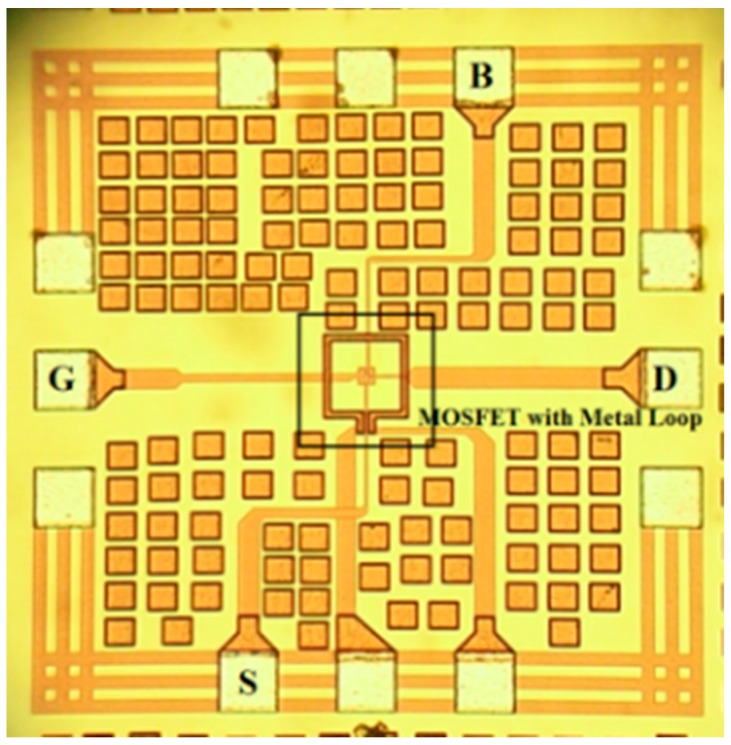
Chip photograph.

The device under test was fabricated using TSMC 0.18 µm standard CMOS process with channel length 0.18 µm and channel width 18 µm, the gate oxide thickness is about 37 Å and the gate material is polysilicon. The chip photograph is shown in Figure 3.

A square metal loop was designed and the device was placed at the center of the loop to get the maximum effect of the created magnetic field in the active area of the device under test (DUT). The DUT is much smaller in dimension than that of the metal loop around it, and also the DUT is placed at the center of the metal loop. So the magnetic field created by the metal loop is considered to be uniform throughout the DUT. The top metal used in the MOSFET is Metal 3 whereas the loop was created using Metal 5. The distance between the metal loop and the DUT is very small. For these abovementioned reasons, the spatial variation of the magnetic field will be negligible on the DUT. On the other hand, the uniform-sized metal loop acts like a small Helmholtz coil. Therefore, the generated magnetic field is considered uniform and the direction of the magnetic field can be controlled precisely, which in turn minimizes the effect of spatial variation of the magnetic field on DUT as well.

**Figure 4:**
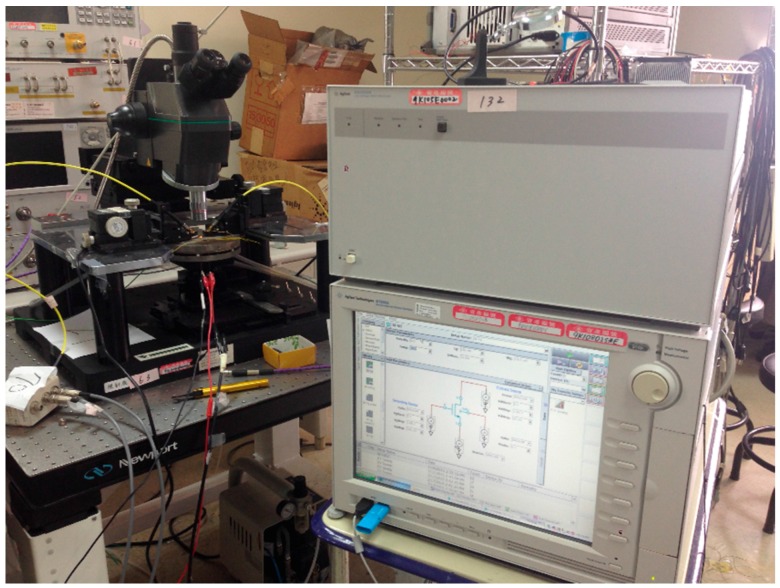
Measurement setup.

Throughout this article all the measurements were performed under direct current (DC) bias, just to ensure that a normal-gate single-drain MOS transistor can be used as a magnetic sensor. Although, as the importance of alternating current (AC) response characteristic measurements are inevitable, so it can be done as the continuation of this work. The measurements were carried out in room temperature on the anti-vibration probe station using Agilent B1500A semiconductor device parameter analyzer as shown in Figure 4. After connecting the DUT with probes in the probe station and wire bonding, it was very hard to shield the DUT using metal, as the available probe station was not shielded. The DUT was placed on top of a surface where the effect of the magnetic field was very low and this was reconfirmed using F. W. Bell 5180 Gauss/Tesla meter so that the presence of electromagnetic interference would be very low around DUT. The gate and drain of the DUT were connected using radio frequency (RF) probes via high-precision current measurement ports of Agilent B1500A to measure the current very precisely. The source, the substrate terminals, and the metal loop were connected using wire bonding methods and via medium-range precision ports from the Agilent B1500A system. However, a proper shielding around the device might provide more accurate results.

**Figure 5:**
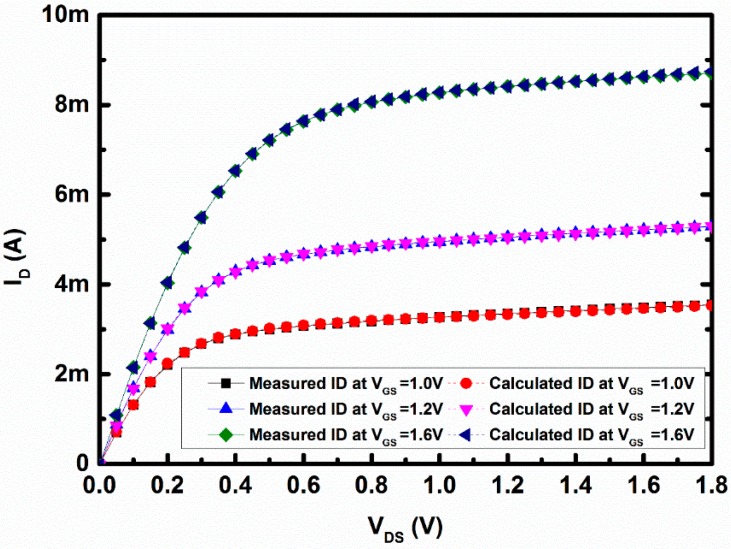
Measured and calculated I_D_–V_DS_ curve at V_GS_ = 1.0 V, V_GS_ = 1.2 V, and V_GS_ = 1.6 V.

Solid lines in Figure 5 represent the I_D_–V_DS_ characteristic for the n-MOSFET structure without the applied magnetic field, where the vertical axis represents drain current (I_D_) in Ampere (A) and the horizontal axis represents the change in drain-to-source voltage (V_DS_). The gate-to-source voltages for these three sets of curve were fixed at 1.0 V, 1.2 V, and 1.6 V, respectively. The basic equations governing carrier flows in the channel of the n-MOSFET are given in Equations (3) and (4), respectively.

(3) $$I_{D}=\;\mu_{n}\frac{\varepsilon_{ox}}{t_{ox}}\frac{W}{L}\;\left[\left(V_{GS}-V_{t}\right)V_{DS}-\frac{1}{2}V_{DS}^{2}\right]\left(1+\lambda V_{DS}\right)$$

(4) $$I_{D}=\;\frac{1}{2}\mu_{n}\frac{\varepsilon_{ox}}{t_{ox}}\frac{W}{L}\;{\left(V_{GS}-V_{t}\right)}^{2}\left(1+\lambda V_{DS}\right)$$

In the above equations, channel width (W) = 18 µm, channel length (L) = 0.18 µm, and dielectric constant ε_ox_ = $3.9\times 8.85\times 10^{-12}$ F/m. From I_D_–V_GS_ plot the threshold voltage (V_t_) has been extracted by curve fitting and its value was 0.32 V. Applying Equations (3) and (4) to the above values and corresponding drain currents, the mobility, oxide thickness ($3.7\times 10^{-9}\;$ m), and short-channel effect parameter lamda (λ) were calculated for each curve to reduce the error. Calculated lamda (λ = 0.0849) and mobility were used to calculate the drain current for the corresponding V_DS_, which ensures nearly 2% error of the measured values. Calculated I_D_–V_DS_ curve from the Equations (3) and (4) was shown in broken lines in Figure 5, where (●) denotes the calculated curve for V_GS_ = 1 V, (▼) represents same for V_GS_ = 1.2 V and (◄) represents same for V_GS_ = 1.6 V. The data in Figure 5 were collected without applying any magnetic field. This set of data was collected to compare whether the calculated data and the measured data were well-matched or not. The substrate current was also measured, but since the DUT was not stressed by the magnetic field, the substrate current was negligible in magnitude, as expected.

**Figure 6:**
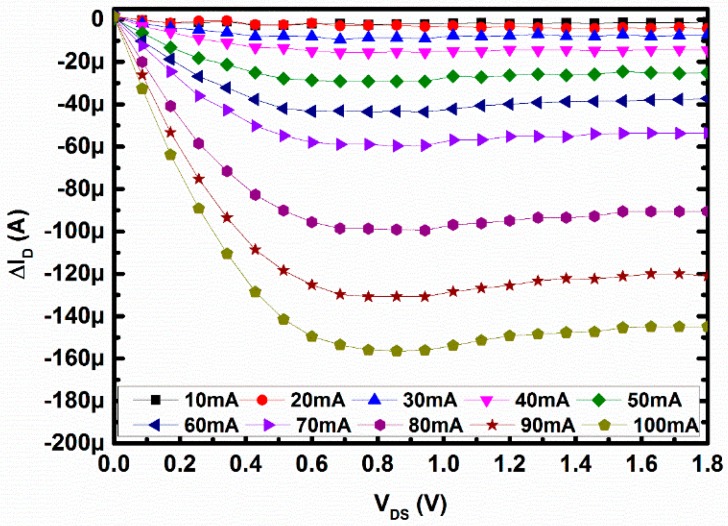
Change in I_D_ versus V_DS_ with different currents through the loop.

To evaluate the change in drain current due to F_B_, ΔI_D_ has been defined as the difference between the drain currents with and without the magnetic field generated by the on-chip loop. From the aforementioned explanation in Section 2, due to the generated magnetic field the effective drain current will decrease; this can be observed in Figure 6. The change of drain current (ΔI_D_) with respect to drain-to-source voltage (V_DS_) has been displayed in Figure 6, in which the horizontal and vertical axes refer to V_DS_ in V and drain current (ΔI_D_) in A. Each curve refers to the difference between current due to no magnetic field and current with the applied magnetic field through the loop from 0–100 mA with the step of 10 mA. During this measurement, the gate-to-source voltage was fixed at 1 V. As seen, the current through the metal loop (i.e., F_B_) was increasing the change in drain current, which was getting higher. This is because whenever F_B_ is getting higher, more mobile carriers are pushed away from the channel, hence the drain current is decreased, and a maximum of 145 µA or 8.06 µA/micron device width at 1.8 V V_DS_, difference current can be measured. The change of drain current at V_DS_ = 1.8 V and V_GS_ = 1 V is around 4.084%. Such change in drain current can be described in terms of either the change in threshold voltage or the change in mobility of charged carriers in the channel. Since the F_B_ was acting horizontally to the channel, the threshold voltage of the device cannot be affected and hence the change in drain current will be ascribed due to the change in mobility of the charged carriers. From the measured I_D_ in the saturation region, the electron mobility variation at different magnetic fields has been derived. The carrier mobility in the channel affected by factors like mobility related to scattering of phonons, screened coulomb scattering, and mobility related to surface roughness scattering. Thus, the increase of the strength of the magnetic field affects the scattering factors effectively, which effectively decreases the mobility of the channel electrons and, as a result, the I_D_ decreases further.

**Figure 7:**
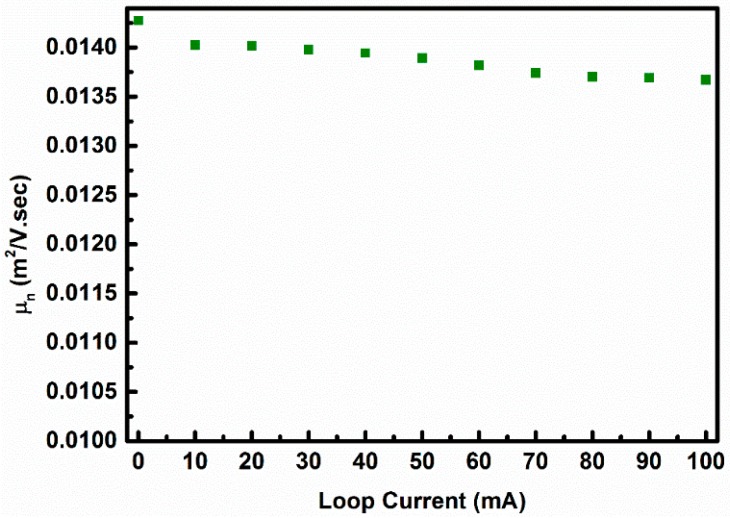
Change in mobility with the applied magnetic field.

The estimated change in mobility with the change in the applied loop current is shown in Figure 7, where the vertical axis represents the change in mobility in m^2^/V·s, whereas the horizontal axis represents the applied current through the metal loop in mA. As can be seen from Figure 7, the effective mobility is decreasing as the current through the loop is increasing, which in turn is decreasing the I_D_ effectively. The change in mobility is around 4.227%.

As shown in Figure 2, due to the Lorentz force the carriers will drift away from channel towards the bulk for both directions of the force, i.e., when the loop current is reversed, electrons from the channel will still drift away towards bulk, but in a different direction as shown in Figure 2b. So the change in mobility is expected to be the same even with the change in direction of the applied loop current. From Figure 7 it can be deduced that the change in mobility is related to the applied loop current, or in other words, to magnetic field, which suggests that the mobility of the charged carriers might be altered by the application of the magnetic field.

**Figure 8:**
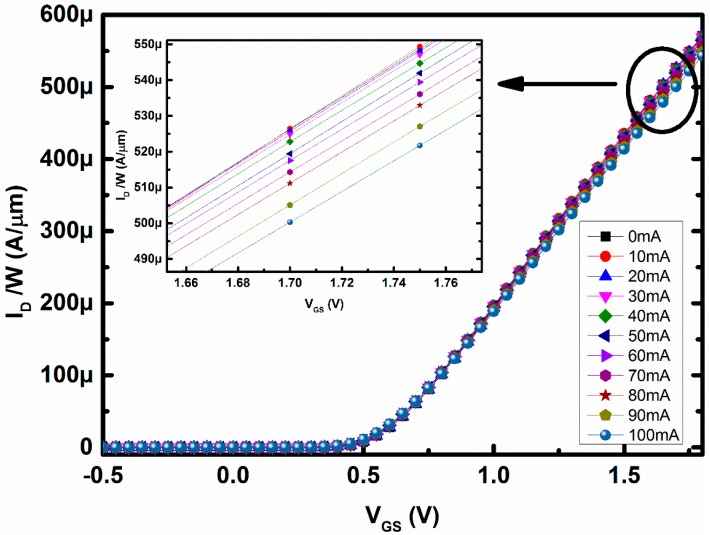
Change in drain current with gate-to-source voltage magnified at 1.5–1.7 V is shown in inset.

**Figure 9:**
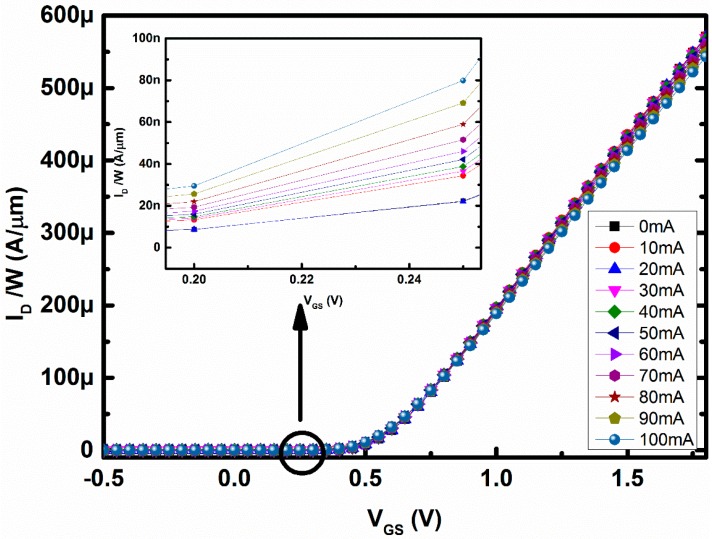
Change in drain current with gate-to-source voltage magnified in 0.2–0.25 V is shown in inset.

Figure 8 shows the relation between drain current per micron width (I_D_/W) and gate-to-source voltage (V_GS_) for different amounts of loop current through the metal loop around the MOSFET and with the V_DS_ fixed at 1 V. In Figure 8, the gate-to-source voltage (V_GS_) was magnified at around 1.5–1.7 V, i.e., the device was operating in the saturation region of operations. It can be seen from Figure 8 that I_D_ was decreasing with increasing magnetic field. In the saturation region, drift was the dominant mechanism for the charged carriers and hence the I_D_ decreases due to the effect of magnetic field. However, when the device operates in the subthreshold regions the change in I_D_ is in the opposite direction, as shown in Figure 9. The applied magnetic field will change the mobility in the subthreshold region, similar to the saturation region of operation. However, the Lorentz force created by the applied magnetic field will change the surface potential, and as a result the threshold voltage of the DUT in the subthreshold region will change as well [20]. Hence, the current will increase irrespective of the decrement of mobility, as the change in threshold voltage is dominant over that of mobility change. However, due to the change in mobility in the opposite direction, the current in the subthreshold region will not be able to increase exponentially, as shown in Figure 9.

**Figure 10:**
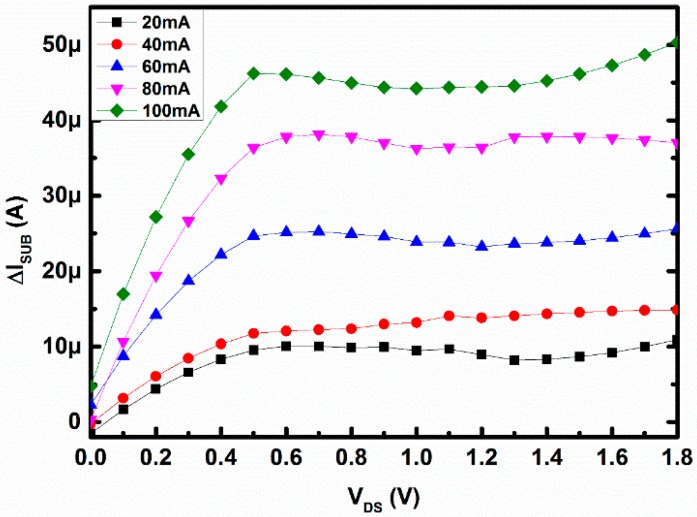
Change in substrate current with V_DS_ for different magnetic field.

During the data collection for Figure 6 there was a significant amount of change in substrate current as shown in Figure 10. From Figure 10 it can be seen that the change in substrate current is lower in magnitude than that of the change in drain current seen in Figure 6. This change arises due to the fact that some of the drifted electrons from the channel might have been lost. Due to recombination in the p-type substrate, the rest electrons will add up to the substrate current, which is shown in Figure 10. As shown in Figure 2, for any direction of the loop current, the drifted-away electrons will go towards substrate only and not towards the gate, hence there was no change in the effective gate current observed. Also, due to the thick gate oxide for 0.18 µm standard CMOS technology, the moved-out electrons will not penetrate towards the gate to build up an effective gate current. In conclusion, as the trend of the change in substrate current is opposite to that of the change in drain current, so the charge neutrality is justified in this case. Figure 10 represents the change in substrate current with V_DS_, whereas the gate voltage was fixed at 1 V as mentioned earlier. These pushed-out electrons might create higher substrate current with increasing magnetic field, as expected, where the vertical axis represents a change in substrate current and the horizontal axis represents the applied drain-to-source voltage in Figure 10. This measurement was done with loop current 0–100 mA. Around 50.24 µA or 2.78 µA/micron device width at 1.8 V V_DS_, difference substrate current has been measured.

**Figure 11:**
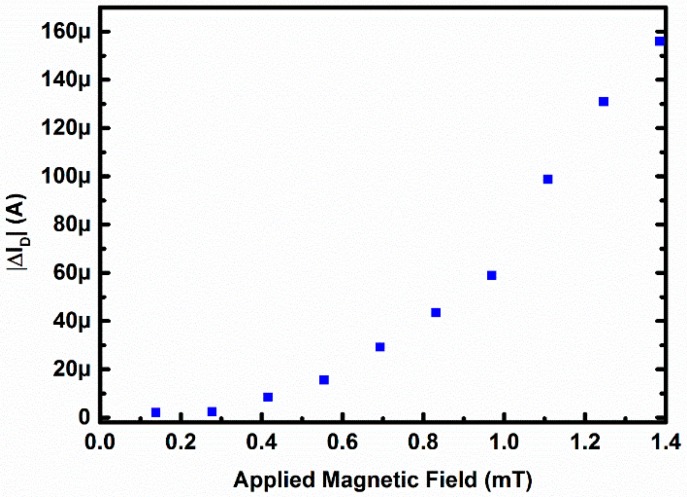
Absolute change in drain current with applied magnetic field.

Figure 11 demonstrates that the MOSFET is directly responding to the magnetic field. The horizontal axis represents the value of the applied magnetic field, where as the vertical axis represents the absolute value of the change in drain current for V_GS_ = 1 V and V_DS_ = 0.8 V. From Figure 11 it can be seen that the absolute change in current is proportional to that of the change in magnetic field.

Finally, the influence of magnetic field over drain current has been measured by defining the sensitivity based on the change of drain current upon the application of the magnetic flux on the charged carriers in the channel of n-MOSFET. The sensitivity can be defined as

(5) $$S=\frac{\Delta I_{D}}{I_{D}}\;.\;\frac{1}{\left|B\right|}$$

where Δ*I_D_* is the difference between the drain current with and without magnetic field and B is the magnetic field strength. The measured value of the generated magnetic field, using F.W.Bell 5180 Gauss/Tesla meter, was very low because the probe of the Gauss meter cannot reach below 1 cm from the chip surface due to probing and wire bonding in the chip. So, to get the value of the applied magnetic field, Equation (2) was used and the calculated value was 1.385 mT. Using the value of the magnetic field, the sensitivity has been calculated and it was about 4.084% (29.6 T^−1^) at V_DS_ = 1.8 V and V_GS_ = 1 V and 3.68% (26.57 T^−1^) at V_DS_ = 0.25 V and V_GS_ = 1 V.

A comparison table (Table 1) is provided to compare this work with previously reported works. In this performance comparison the proposed idea has been compared in the view of sensitivity, channel length, strength of applied magnetic field, device type, etc. For this work, TSMC 0.18 µm standard CMOS technology was used to fabricate the DUT. The dimension of the device is much smaller than that of the metal loop area and also the DUT is placed at the center of the loop. The metal loop is made up of Metal 5, i.e., the distance of the metal loop is very small from that of the DUT. So the created magnetic field is considered to be uniform, and the magnetic field is expected to be uniform throughout the DUT due to its small size. So, to compute the magnetic field strength the method was used as proposed in [19]. This was a rectangular-shaped normal MOSFET with normal gate and single drain. The maximum applied magnetic field was 1.385 mT. The estimated sensitivity was 4.084% or 29.6 T^−1^, which suggests that the proposed structure was not only very effective but also more sensitive with respect to the proposed work. As reported in [12], the sensitivity was related to channel length, mobility, and applied magnetic field. So, keeping mobility and applied magnetic field constant, if long channel devices could be used, the sensitivity will increase more and the device will be more sensitive to detect the magnetic field. However, the above results lead us to report that the short-channel single-drain normal-gate CMOS transistor can also be used as a magnetic sensor with compatible sensitivity. Also, a long-channel DUT might suffer from spatial variation along the channel of the DUT due to the long-channel dimension. In this article, a wide-gate NMOS transistor is used to ensure easier measurements. In a wide transistor, high current can be expected, and hence it will be easier to get the difference current. Although, this proposed magnetic sensor has single gate, drain, and source, i.e., this DUT is a common MOSFET and not a special device like MAGFET or others. However, these kinds of sensors can suffer from issues like non-idealities, which can be solved by using proper readout circuit design techniques like chopping-spinning technique [15].

## 4. Conclusions

The drain current modulation of a single drain normal gate n-MOSFET has been carried out under the influence of a small magnetic field generated by the on-chip metal loop. Due to the applied magnetic field on the inversion layer of the n-MOSFET, a portion of mobile charged carriers was pushed out of the channel and the drain current was reduced. As much as 145 uA (8.06 uA/per micron device width) difference current was detected under 1.385 mT magnetic fields. To counter the drain current, the change in the substrate current was also measured, although it is lower in magnitude than that of the difference drain current due to recombination loss in the substrate region. The effective change in mobility due to the applied magnetic field has also been examined, and 4.227% change in mobility was obtained. The I_D_–V_GS_ plot reflects different behaviors in subthreshold and strong inversion region under the influence of Lorentz force. Finally, the sensitivity of the proposed device has been measured accordingly based on the difference current due to the on-chip magnetic field and the maximum 4.084% (29.6 T^−1^) sensitivity was estimated. Therefore, this single-drain normal-gate n-MOSFET has been verified as suitable for magnetic sensing applications.

## Figures and Tables

**Table 1:** Comparison with the previously reported work.

Ref.	Dev Type	Channel Length (µm)	Application of Magnetic Field	Device Structure	Maximum Strength of Applied Magnetic Field (mT)	Sensitivity (T^−1^)	Process
[16]	MOSFET	48	Off-chip	Offset trimmable MAGFET array (dual drain)	100	2–4%	2.4 um CMOS
[13]	MOSFET	48	Off-chip (Helmholtz coil)	Rectangular & sector (dual drain)	1000	2.84 max for thick oxide sector devices	0.18 um CMOS
[14]	MOSFET	80	On-chip	Rectangular & Sector (dual drain)	-	1%	1.0 um BiCMOS
[17]	Pt/MZF/YSZ MOSFET	10	Off-chip	Rectangular (single drain)	600	<0.1%	
**This work**	**MOSFET**	**0.18**	**On-chip**	**Rectangular (single drain)**	**1.385**	**4.08% (29.6 T^−1^)**	**0.18 um CMOS**
